# Computed Tomographic Evaluation of the Sagittal Ridge of the Third Metacarpal Bone in Young Thoroughbred Racehorses: A Longitudinal Study

**DOI:** 10.3390/ani14050812

**Published:** 2024-03-06

**Authors:** Koppány Boros, Sue Dyson, Ágnes Kovács, Zsolt Lang, Annamaria Nagy

**Affiliations:** 1 Department and Clinic of Equine Medicine, University of Veterinary Medicine Budapest, Dóra Major, 2225 Üllő, Hungary; nagy.annamaria@univet.hu; 2 The Cottage, Church Road, Market Weston, Diss IP22 2NX, UK; sue.dyson@aol.com; 3 Department of Biostatistics, University of Veterinary Medicine Budapest, 1078 Budapest, Hungary

**Keywords:** racehorse, fetlock, computed tomography, third metacarpal bone, sagittal ridge, osteochondrosis, adaptive remodelling

## Abstract

**Simple Summary:**

Pain in the fetlock region is a common cause of lameness in racehorses. Radiological abnormalities of the sagittal ridge of the cannon bone have been previously linked to joint effusion, lameness and reduced sales prices. However, to date, no data exist on the development and progression of these lesions. The aim of this study was to describe the computed tomographic appearance of the sagittal ridge in young racehorses and to document the progression of the detected findings over three assessments in the first year of training and racing. At the first examination, 40 yearlings underwent computed tomographic examination of both front fetlocks. Re-examinations were performed twice, approximately six months apart on 31 and 23 horses, respectively. Computed tomographic recordings were analysed with particular attention to changes in bone density and to lesions consistent with osteochondrosis (developmental abnormality). An increase in bone density of the sagittal ridge occurred in the first six months of training. Lesions consistent with osteochondrosis could decrease in size or resolve during the first year of training. In this population of racehorses these changes were not associated with lameness.

**Abstract:**

Metacarpophalangeal joint region pain is a common cause of lameness in racehorses. Radiological abnormalities in the sagittal ridge (SR) of the third metacarpal bone have been associated with joint effusion, lameness and reduced sales prices. The aims were to describe computed tomographic (CT) appearance of the SR in racehorses, and to document the progression of these findings over three assessments. Forty yearlings were enrolled at the first examination (time 0). Re-examinations were performed twice, approximately six months apart on 31 (time 1) and 23 (time 2) horses, respectively. Computed tomographic examinations of both metacarpophalangeal regions were performed with the horses in a standing position. Computed tomographic reconstructions were analysed subjectively and objectively. The mean Hounsfield Unit values (Hus) of eight radial segments and location, size and shape of hypoattenuating lesions were recorded. Mean Hus at time 1 were higher than at time 0. There was no difference between mean HU at times 1 and 2. The mean HU values of the dorsal half were higher in the right forelimbs and in fillies. Hypoattenuation was identified in 33/80 (41.3%) limbs at time 0, in 22/62 (35.5%) limbs at time 1 and in 14/46 (30.4%) limbs at time 2. All hypoattenuations were located in the dorsodistal aspect of the SR. The most common shapes were hypoattenuating lesions elongated proximodistally and those extending towards trabecular bone. An increase in attenuation of the SR occurred in the first six months of training. Hypoattenuating lesions could decrease in size and could resolve during early training. In this population, these lesions were not associated with lameness.

## 1. Introduction

Pain in the metacarpophalangeal joint region is one of the most common causes of lameness in Thoroughbred racehorses [1]. Radiographic abnormalities in the sagittal ridge of the third metacarpal bone have been described and associated with mild-to-moderate joint effusion, lameness and reduced sales prices [2,3]. In previous studies, the prevalence of radiographic abnormalities of the sagittal ridge in Thoroughbred yearlings varied between 7.5% and 60.8% [4,5,6,7,8]. These abnormalities included radiolucent areas of varying shape and size, flattening of the dorsal contour of the sagittal ridge and a separate radiopacity at the dorsoproximal aspect of the sagittal ridge consistent with fragmentation [9]. Most sagittal ridge lesions are best detected in lateromedial (flexed) radiographs [9]. The aetiopathogenesis of sagittal ridge lesions is debated; developmental and traumatic origins have both been hypothesised [1]. The frequent bilateral presence suggests that most sagittal ridge changes are developmental in origin and are generally believed to be a form of osteochondrosis [8], which is a disorder caused by focal disturbance in endochondral ossification. In the early stages, the disease is only visible histologically and is confined to the growth cartilage (osteochondrosis latens); as the disorder progresses, it becomes macroscopically and radiographically detectable (osteochondrosis manifesta); and in advanced cases cartilage flaps can detach from the parent bone (osteochondrosis dissecans) [10]. In forelimbs, the dorsal aspect of the sagittal ridge is considered to be the most common site of osteochondrosis [1].

Computed tomography (CT) of the distal aspect of the limb of standing sedated horses is becoming increasingly available worldwide [11]. Three-dimensional visualization of anatomical structures allows precise localisation and characterisation of abnormalities and facilitates objective measurements. Subjective computed tomographic, magnetic resonance imaging and radiographic findings in the metacarpophalangeal joint of Thoroughbred yearlings have recently been described [12]. Abnormalities in the sagittal ridge included hypoattenuating lesions and increased attenuation of the subchondral and/or trabecular bone. Longitudinal data are available on the change of subchondral bone density at predilection sites in the metacarpophalangeal joints of young Thoroughbred racehorses, using cone-beam CT [13]. However, this study did not consider abnormalities of the sagittal ridge. To date, computed tomographic abnormalities of the sagittal ridge have not been described in detail, and to our knowledge, there is no longitudinal information on the progression of sagittal ridge lesions in young Thoroughbreds. 

The aims of this study were to describe the computed tomographic appearance of the sagittal ridge in young Thoroughbred racehorses, using subjective and objective evaluations, and to document the progression of these findings over three assessments in the first year of training and racing. We hypothesised that 1. the mean attenuation of the sagittal ridge would increase during the study period, as an adaptative response to training; and 2. the appearance of some hypoattenuating lesions in the subchondral bone of the dorsal aspect of the sagittal ridge would change during the first year of training.

## 2. Materials and Methods

Forty yearling Thoroughbred racehorses were enrolled in the study. All horses were born in the previous year and were entering flat race training. Hungarian racehorse owners and trainers were invited to participate in the project. Participants were selected in order of application. Horses were eligible if they were free from lameness and had no history of metacarpophalangeal joint disease. Signalment of horses (sex, weight, height at withers, age in days), training history (number of days/weeks spent in training, number of fast-work days/week, average fast work distance, average distance of canter training/day, any period of >7 days without training, any injury/lameness, date and result of races, direction of training and racing) were collected. To assess potential associations with cumulative exercise, horses were grouped based on the highest intensity of training achieved prior to each examination: no training; light training, consisting of walking and trotting exercises; training without any fast work; training including fast work. 

The first examination (time 0) was performed towards the end of yearling age in all horses. Re-examinations (times 1 and 2) were performed twice, approximately six months apart. Each examination included a clinical assessment, paying particular attention to the metacarpophalangeal region. The presence of the following findings was recorded for each forelimb: distension of the metacarpophalangeal joint capsule, diffuse or localised swelling and/or heat in the metacarpophalangeal region, pain on palpation or on manipulation, and restricted range of passive motion. Subjective and objective (Equinosis Q Lameness Locator^®^, Columbia, SC, USA) lameness examinations were performed. For the subjective evaluation a numerical grading system, ranging from 0 to 8 [14], was used. At time 0, lameness evaluation was limited to walking and trotting in a straight line on a hard surface. At times 1 and 2, distal forelimb flexion tests and gait evaluation on the lunge/led in a circle on soft and hard surfaces, in trot, were also performed if permitted by the horse’s behaviour. If a horse showed forelimb lameness at time 1 or 2, diagnostic anaesthesia was performed to localise the source of pain causing lameness.

Computed tomographic examinations of both metacarpophalangeal regions were performed with the horses in a standing position. Horses were sedated with a combination of detomidine hydrochloride (0.01 mg/kg IV, Domosedan, Orion Pharma, Budapest, Hungary) and butorphanol (0.02 mg/kg IV Nalgosed, Bioveta, Ivanovice na Hané, 683 23, Czech Republic). The CT examinations were performed with a 16-detector multislice helical scanner (Qualibra^®^ Canon Aquilion LB^®^). Each limb was scanned from the distal third of the metacarpal region to at least the junction of the proximal and distal halves of the proximal phalanx. Acquisition values were 135 kV and 350 ms, 300 mm range, 300 mm field of view, 0.5 mm slice thickness and 0.5 s rotation time. Computed tomographic images were analysed in a medical image viewing software (JiveX DICOM Viewer^®^ Version 5.2, Visus Health IT GmbH, Bochum, Germany) in multiplanar reconstruction, using the bone algorithm and 0.3 mm extension voxel size. 

**Figure 1:**
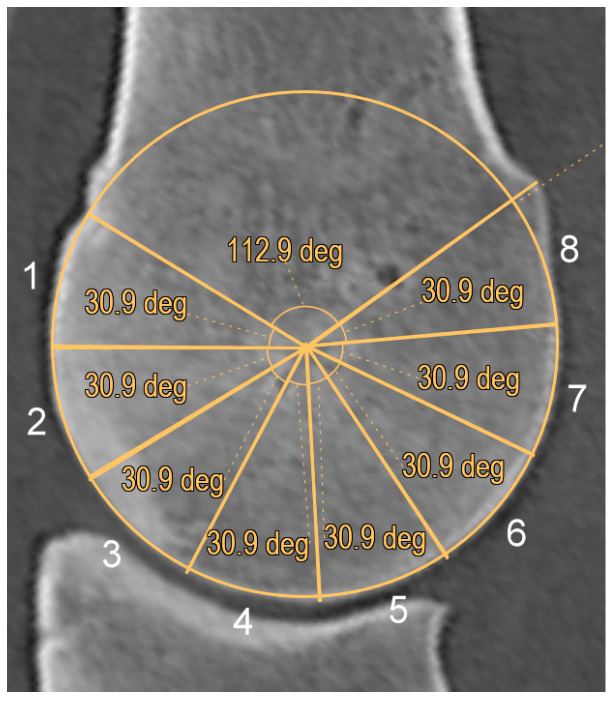
Sagittal computed tomographic reconstruction of the sagittal ridge of the third metacarpal bone (dorsal is to the left) showing the eight radial segments of the sagittal ridge, in which mean Hounsfield Unit values were recorded. The segments were categorized into dorsal (1–4) and palmar (5–8) halves.

Subjective and objective image analyses were performed. A sagittal plane reconstruction was set transecting the most dorsal and palmar aspects of the sagittal ridge. In this image, a circle was aligned to the outline of the sagittal ridge, as precisely fitting as possible. The dorsoproximal and palmaroproximal extents of the sagittal ridge were connected to the centre of the circle, and the sagittal ridge was divided into eight radial segments of the same angle. The eight segments were categorized and also pooled in dorsal (1–4) and palmar (5–8) halves (Figure 1). The mean Hounsfield Unit (HU) values of the whole sagittal ridge, the dorsal and palmar halves and in each segment representing separate region of interests (ROI) were recorded. Limbs were grouped as right or left in order to investigate any laterality in mean HU values.

**Figure 2:**
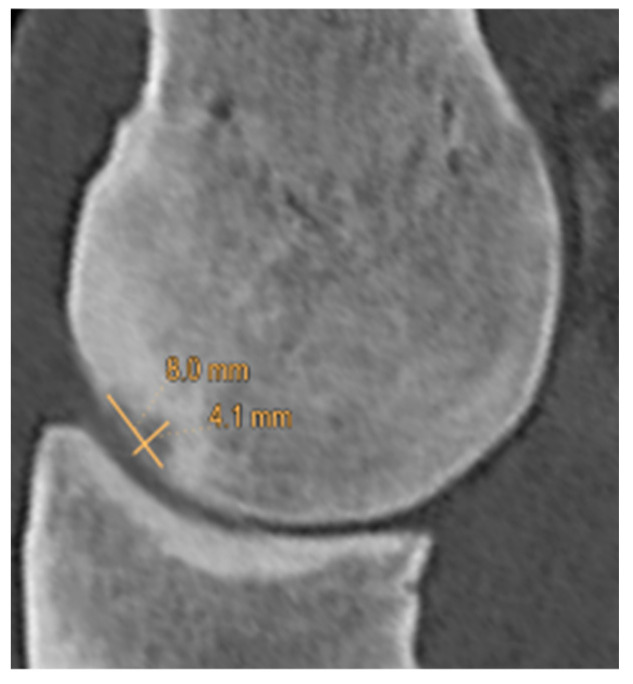
Sagittal computed tomographic reconstruction of the sagittal ridge of the third metacarpal bone (dorsal is to the left) showing measurements of the proximodistal (8.0 mm) and dorsopalmar (4.1 mm) extents of a hypoattenuating lesion extending towards trabecular bone. Note the hyperattenuating area surrounding the lesion.

**Figure 3:**
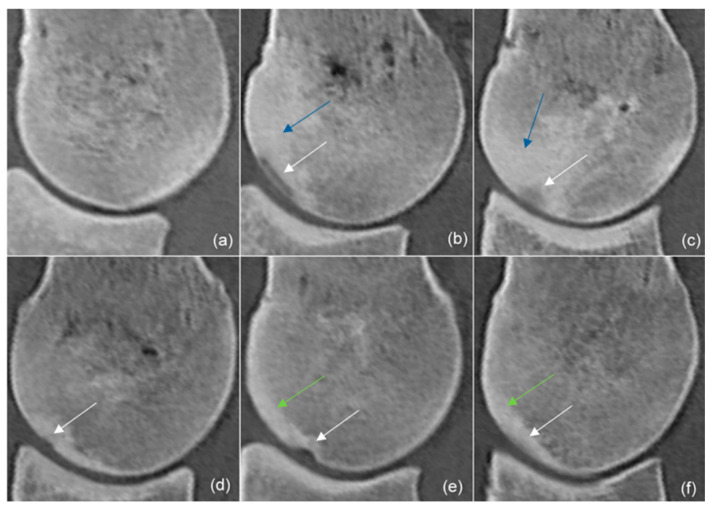
Sagittal computed tomographic reconstructions of the sagittal ridge of the third metacarpal bone (dorsal is to the left): a normal dorsal contour (**a**) and different shapes of hypoattenuating lesions (white arrows) in the subchondral bone of the sagittal ridge: proximodistally elongated lesions: the proximodistal extent of the lesion exceeds the dorsopalmar extent (**b**), lesions extending towards trabecular bone: the dorsopalmar extent of the lesion exceeds the proximodistal extent (**c**), uneven depressions: a depression in the subchondral bone surface with uneven margins (**d**), indentations: a depression in the subchondral bone surface with even margins (**e**), subtle lesions: minor hypoattenuation that did not fit the description of any of the categories above (**f**). Note the hyperattenuating area (blue arrow) surrounding the hypoattenuating lesion (**b**), the cone-shaped hyperattenuation (blue arrow) in the trabecular bone extending from around the hypoattenuating lesion, occupying more than half of the dorsopalmar extent of the bone (**c**), and the thickening of the subchondral bone plate (green arrow) (**e**,**f**).

The presence, mediolateral location and proximodistal position of hypoattenuating lesions were recorded in each segment. The largest proximodistal and dorsopalmar extents of the hypoattenuating lesions were measured on sagittal plane reconstructions aligned to the lesion (Figure 2). The appearance of hypoattenuating lesions on the sagittal plane reconstructions was described as: 1. proximodistally elongated lesions (if the proximodistal extent of the lesion exceeded the dorsopalmar extent), 2. lesions extending towards trabecular bone (if the dorsopalmar extent of the lesion exceeded the proximodistal extent), 3. uneven depressions (a depression in the subchondral bone surface with uneven margins), 4. indentations (a depression in the subchondral bone surface with even margins), 5. subtle lesions (minor hypoattenuation that did not fit the description of any of the categories above) (Figure 3).

**Figure 4:**
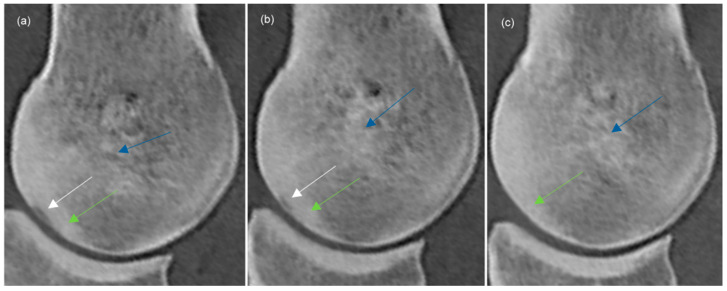
Sagittal computed tomographic reconstructions of the sagittal ridge of the third metacarpal bone (dorsal is to the left) of the same metacarpophalangeal joint showing a hypoattenuating lesion (white arrows) in the subchondral bone of the sagittal ridge detected at the initial examination, time 0 (**a**) that decreased in size at the first re-examination (time 1) (**b**) and was no longer detectable at the second re-examination (time 2) (**c**). Note the thickening of the subchondral bone plate (green arrows) (**a**–**c**) and patchy hyperattenuation in the trabecular bone (blue arrows) (**a**–**c**).

Thickening of subchondral bone was recorded. The subchondral bone plate was considered thickened if it did not run as an even line parallel to the dorsal outline of the sagittal ridge (Figure 3e,f). The appearance of hyperattenuation in trabecular bone was recorded as being cone-shaped or patchy (Figure 3c and Figure 4). A repeatability study was carried out obtaining all measurements three times on three limbs. The coefficient of variance was calculated using Microsoft Excel (Microsoft Corporation One Microsoft Way, Redmond, WA 98052-6399 USA) with a result of <2% for all variables.

Descriptive data analysis was performed on the location, shape and size (median, 95% confidence interval) of the hypoattenuating lesions of the sagittal ridge; on the age of horses (mean, ±standard deviation); on the time since previous examination (mean, ±standard deviation); on the sex and training/racing history of horses. All statistical analyses were carried out using the statistical programming environment R [15]. A generalized linear mixed effects model [16] was used to investigate the mean HU values calculated separately for the dorsal half (segments 1–4) and palmar half (segments 5–8). To implement this, the lme() function from the nlme package [17,18] in R was applied. For both dorsal HU values and palmar HU values the fixed factors were time of examinations (0, 1, 2), limb (right, left), sex of the horse (filly, colt), baseline age of the horse, bodyweight, height at withers and hypoattenuation (yes, no). Additionally, Thoroughbred racehorses were included as random subjects. The differences between the means at each round of examinations were also calculated using the generalized linear mixed effects model mentioned above. Subsequently, adjusted *p*-values were calculated for Tukey’s pairwise multiple comparisons. The glht() function of the R multcomp package [19] was used to perform the analysis. Statistical significance was set at *p* ≤ 0.05.

## 3. Results

### 3.1. Horses

A total of 40 Thoroughbred yearlings (26 colts and 14 fillies) from ten racehorse trainers were included at time 0. Signalment and training history of horses at each examination is shown in Table 1. Prior to the initial examination, 27/40 (67.5%) horses had been engaged in some form of training: 26 were exercised in trot (up to 30 min a day and up to seven times a week) and one also in canter (for four weeks). No horse had carried out any fast work. At time 1, 31 horses were examined. Nine horses had been trained in canter without any fast work and 22 horses had been engaged in fast speed training. Three fillies had raced once. At time 2, 23 horses were examined. Preceding this, 19/23 (82.6%) horses had raced (once n = 4, twice n = 6, three times n = 6, and four times, n = 3). Fillies had a significantly higher number of starts compared with colts (*p* = 0.003). Two horses were trained in canter without any fast work and 21 horses were engaged in fast work training prior to time 2.

During the study period only one horse showed pain causing lameness localised to the metacarpophalangeal region due to an apical fracture of the left fore medial proximal sesamoid bone and injury of the medial suspensory ligament branch three months prior to time 2.

### 3.2. Hounsfield Unit Measurements

Mean HU values at time 1 were significantly higher in both the dorsal (*p* < 0.001) and palmar (*p* < 0.001) halves of the sagittal ridge than those measured at time 0 (Table 2). There was no significant difference between mean HU values of the dorsal (*p* = 0.789) and palmar (*p* = 0.853) halves measured at times 1 and 2. The mean HU value of the dorsal half of the sagittal ridge was significantly higher than the mean for the palmar half (*p* = 0.023). Mean HU values of the dorsal half were significantly higher in right forelimbs than in left forelimbs (*p* < 0.001). Mean HU values of the dorsal half of fillies were significantly higher than mean HU values of the dorsal half of colts (*p* = 0.037). There were no significant differences in mean HU values between the right and left forelimbs (*p* = 0.082) and between colts and fillies in the palmar half (*p* = 0.085). 

There was a no significant (*p* = 0.118) decrease in total HU value of the whole sagittal ridge in one limb of one horse between times 0 and 1, and in 23 limbs of 14 horses between times 1 and 2. All of these horses except one had already carried out fast-speed work prior to examination. 

### 3.3. Hypoattenuating Lesions

A hypoattenuating lesion in the subchondral bone of the sagittal ridge was identified in 33/80 (41.3%) limbs at time 0; in 14 horses bilaterally (Table 3). At time 1, a hypoattenuating lesion was identified in 22/62 (35.5%) limbs; in eight horses bilaterally. No new lesions, that could not be detected at time 0, were identified. At time 2, a hypoattenuating lesion was seen in 14/46 (30.4%) limbs; in five horses bilaterally. No new lesions that had not been detected at time 1 were identified. In horses with ≥2 examinations, in six limbs of four horses, the hypoattenuating lesion was no longer present at time 1; at time 2, the hypoattenuating lesion was not identifiable in a further five limbs of four horses (Figure 4). During the study period, no progression in the size of any of the lesions was detected.

All hypoattenuating lesions were located in the dorsodistal aspect of the sagittal ridge. Only the second, third and fourth segments of the sagittal ridge were involved. At time 0, 18/33 (54.5%) lesions affected only the third segment, 1/33 (3.0%) lesion was only in the fourth segment, 9/33 (27.3%) lesions were present in both the second and third segments, 4/33 (12.1%) lesions were present in both the third and fourth segments, and 1/33 (3.0%) lesion involved all three segments. At time 1, 16/22 (72.7%) lesions affected only the third segment, 4/22 (18.2%) lesions were present in both the second and third segments, and 2/22 (9.1%) lesions were in both the third and fourth segments. At time 2, 8/14 (57.1%) lesions affected only the third segment, 4/14 (28.6%) lesions were present in both the second and third segments, and 2/14 (14.3%) lesions involved both the third and fourth segments. At time 0, in 21/33 (63.6%) limbs the hypoattenuating lesion was located in the axial region of the sagittal ridge, in 10/33 (30.3%) limbs in the lateral aspect and in 2/33 (6.1%) limbs in the medial aspect of the sagittal ridge. Of the lesions that resolved over time (n = 11), seven were located in the axial region of the sagittal ridge, three in the lateral aspect and one in the medial. The two most common shapes were proximodistally elongated hypoattenuating lesions and hypoattenuating lesions extending towards the trabecular bone. 

### 3.4. Subchondral Bone Thickening, Trabecular Bone Hyperattenuation

Thickening of the dorsal subchondral bone of the sagittal ridge (Figure 3 and Figure 4) was detected in 74/80 (92.5%) limbs at time 0, in 60/62 (96.8%) limbs at time 1 and in 45/46 (97.8%) limbs at time 2. Hyperattenuation in trabecular bone was detected with and without simultaneous presence of a hypoattenuating lesion in the subchondral bone. Cone-shaped hyperattenuation (Figure 3) was present in 5/80 (6.3%) limbs at time 0, in 7/62 (11.3%) limbs at time 1 and in 5/46 (10.1%) limbs at time 2. Hypoattenuating lesions surrounded by cone-shaped hyperattenuating zones had not resolved by the end of the study period. Patchy hyperattenuation in the trabecular bone (Figure 4) was detected in 14/80 (17.5%) limbs at time 0, in 21/62 (33.8%) limbs at time 1 and in 24/46 (52.2%) limbs at time 2.

## 4. Discussion

This is the first study to provide detailed description and longitudinal information on the progression of computed tomographic abnormalities of the sagittal ridge in young Thoroughbred racehorses.

We hypothesised that 1. the mean attenuation of the sagittal ridge would increase during the study period, as an adaptative response to training, and 2. the appearance of some hypoattenuating lesions in the subchondral bone of the dorsal aspect of the sagittal ridge would change during the first year of training. In agreement with our hypothesis, there was an increase in the mean HU value in the entire sagittal ridge, consistent with increased bone density, in the first year of training in young Thoroughbreds. This is also in agreement with the results of other studies which demonstrated that within a few months following the beginning of training, density of the distal regions of the third metacarpal bone increased due to adaptive remodelling [13,20,21,22]. This physiological response is necessary for the bone tissue to withstand the repetitive strain of training [23]. Racehorses in training have an average 36.8% higher density of the distal metacarpal epiphysis than horses not in training [24].

A significant Increase In mean HU values of the sagittal ridge was measured by the end of the six months interval between times 0 and 1. More pronounced adaptive increase in bone density may occur in the first than in the second half of the first year in training of young Thoroughbreds. This is in agreement with the results of a previous study investigating changes in subchondral density in the metacarpophalangeal joints of young Thoroughbred racehorses [13]. In the current study, in most horses the workload was increased more abruptly (from light training to fast work) between times 0 and 1 than before time 0, while by time 2 most horses had been engaged in fast work.

We hypothesized that HU values would increase throughout the whole study period; however, no significant increase in mean HU values was detected at time 2 compared with time 1. In addition, in some horses the total mean HU value of the sagittal ridge decreased between times 1 and 2. The majority of these horses (61%) were in light training at time 2, most due to their winter rest period. This is consistent with the density of the distal aspect of the third metacarpal bone being higher in racehorses in training than in horses having a period of rest [25]. 

In the current study, the mean HU value of the dorsal half of the sagittal ridge was significantly higher than the mean for the palmar half (*p* = 0.023). This is in contrast to the results of a study which evaluated CT reconstructions in the plane of the parasagittal grooves and the metacarpal condyles, in which there was an increase in density of the distal epiphysis of the third metacarpal bone, as a result of training, extending from the palmarodistal aspect to the dorsoproximal direction, leaving the dorsodistal aspect the least dense [24]. The different results of the two studies may reflect the biomechanical loads sustained by the regions investigated. The subchondral bone of the palmar aspect of the condyles of the third metacarpal bone is compressed by the proximal sesamoid bones [24], whereas the sagittal ridge does not sustain substantial compressive load.

A previous study found no significant difference In the microstructure of the distal aspect of the third metacarpal bone between the left and right forelimbs of active flat racing Thoroughbreds from two racetracks in Ontario, USA [26]. Our results demonstrated significantly higher mean HU values of the dorsal half of the sagittal ridge in right forelimbs than in left forelimbs (*p* < 0.001). During gallop, the leading forelimb experiences the highest magnitude of loading and is the most susceptible to overloading [27,28,29,30,31]. Horses participating in this study were equally trained on both reins; however, because horses run clockwise with the right forelimb leading at Hungarian racetracks, many of the horses had carried out their fast work only on the right rein. This could explain the differences measured between the forelimbs at times 1 and 2. However, this difference was present at time 0 when the horses were just entering training without any laterality in work pattern. The reason for this is unknown. Our data showed significantly higher mean HU values of the dorsal half in fillies than in colts (*p* = 0.037). The significantly higher starts of fillies compared to colts could provide one possible explanation at times 1 and 2. However, this difference was present at time 0 when the horses had not raced yet. The reason for this is unknown.

This is the first study to document longitudinal data on hypoattenuating lesions detected with computed tomographic examination in the sagittal ridge of the third metacarpal bone of horses. These findings may be consistent with osteochondrosis [4,5]; however, histological evaluation would be needed to confirm this. On radiographs osteochondrosis of the sagittal ridge can be detected as flattening, depression or lucency of the dorsal contour [9]. Such radiographic changes were reported with a wide range of frequency of occurrence (4.8–37.1%) in Thoroughbred yearlings [3,5,6], often (39–53%) bilaterally [3,5,6]. The large range among different studies can be related to different populations of racehorses and to different radiographic views obtained to assess sagittal ridge changes. In most studies, only standard radiographic views and lateromedial (flexed) images were examined. However, in a recent study [12], dorsopalmar (flexed) and dorsoproximal-dorsodistal (flexed) (‘skyline’) views of the metacarpophalangeal joints were included in the radiographic protocol, revealing hypoattenuating lesions which were not identified in standard views. The use of digital radiography [12] compared with conventional radiography used in earlier studies [5] may have facilitated increased detection of radiolucent lesions.

Computed tomography allows three-dimensional visualisation; thus, superimposition of anatomical structures can be avoided [13,32,33]. At the initial examination, hypoattenuating lesions were detected with greater frequency than the highest reported numbers of sagittal ridge radiolucent lesions in yearling pre-sale radiographs. It is likely that not all lesions detected in CT images are identifiable radiographically. In a related study that compared computed tomographic and radiographic findings at time 0, 76% of sagittal ridge hypoattenuating lesions were identified radiographically [12]. 

Osteochondrosis of the sagittal ridge was previously associated with lameness in young (≤3 years old) Thoroughbreds [9] and in a mixed population of mostly young (<2.5 years old) Warmblood horses [2]. Diagnostic anaesthesia was not performed in every case to verify the source of pain causing lameness; however, the presence of joint effusion and reduced range of motion supported the clinical diagnosis. In addition to the sagittal ridge lesions in some horses there were other radiological lesions that could have been the cause of lameness. In the current study, only one horse had pain causing lameness localised to the metacarpophalangeal region, but unrelated to the sagittal ridge; therefore, hypoattenuating lesions in the sagittal ridge were not associated with lameness in this group of young Thoroughbred racehorses. 

Osteochondrosis lesions have the potential to heal depending on various factors in the first year of life [34]. Radiological studies investigating the development of equine osteochondrosis established that in the hock, osteochondrosis of the intermediate ridge of the tibia and osteochondrosis of the distal aspect of the lateral trochlea of the talus can resolve in the first five month of life [35]. In the stifle, osteochondrosis of the midregion of the lateral femoral trochlea can resolve in the first 8 months of life [35]. In a radiographic study, osteochondrosis lesions of the third metacarpal bone appeared, disappeared or evolved between the age of 12 and 36 months in Warmblood sports horses [36]. This is in agreement with our study which demonstrated that osteochondrosis lesions of the sagittal ridge of the third metacarpal bone have the potential to resolve even in two-year-old Thoroughbreds.

Some of these lesions are surrounded by a cone-shaped hyperattenuating zone, representing remodelling of trabecular bone [12]. The evolution of hypoattenuating lesions in the subchondral bone of the sagittal ridge with and without trabecular remodelling needs further investigation. These lesions can occur in various shapes on the dorsodistal aspect of the sagittal ridge. Longer term follow-up is required to assess the long-term clinical significance of sagittal ridge lesions during and after racehorse training.

Our study had certain limitations. Lameness assessments at time 0 were not comprehensive in every case because some yearlings were unhandled at the time. Some horses were lost to follow-up. No post-mortem histological examinations were performed; therefore, the aetiopathogenesis of the hypoattenuating lesions could not be determined. Results may not be representative of all racehorse populations since they were collected from a relatively small geographical area. Some results could be influenced by differences in training regimes and the low sample size, particularly when investigating the effect of sex.

## 5. Conclusions

A significant increase in attenuation of the sagittal ridge, reflecting an increase in bone mineral density due to adaptive remodelling, occurred in the first six months of racehorse training. In the study population of young Thoroughbred racehorses, the dorsal half of the sagittal ridge had greater hyperattenuation than the palmar half. Hypoattenuating lesions in the dorsal aspect of the sagittal ridge have a potential to decrease in size and to disappear during the first year of training. In this population of young Thoroughbred racehorses, these lesions were not associated with lameness.

## Figures and Tables

**Table 1:** The signalment, training and racing history of 40 Thoroughbred racehorses which entered training, 31 and 23 of which were re-examined, respectively, at intervals of approximately six months. Training level refers to the cumulative work achieved prior to each examination. n—number of horses, SD—standard deviation.

	Time 0 (n = 40)	Time 1 (n = 31)	Time 2 (n = 23)
**Sex**			
Colt	26	19	12
Filly	14	12	8
Gelding	0	0	3
**Age in days**			
Mean	613.4	803.0	1000.3
±SD	38.0	47.2	56.1
**Time since previous examination in days**			
Mean	-	193.0	200.7
±SD	-	15.4	18.8
**Training level**			
Not in training	13	0	0
Walk and trot	26	0	0
Canter	1	9	2
Fast-speed work	0	22	21
**Raced**	0	3	19

**Table 2:** Model adjusted estimates and 95% confidence intervals for the difference in mean Hounsfield Unit (HU) values between times of examination (initial = 0; first re-examination = time 1; second re-examination = time 2). *p*-values are adjusted for Tukey’s multiple comparisons. There was a significant increase in the mean dorsal HU values for times 1 and 2 compared with time 0. The mean difference between times 1 and 2 was not significant. Similar results were also seen for the palmar HU values. CI—confidence interval.

	Mean Hus Dorsal			Mean Hus Palmar		
	Estimate	95% CI	*p*-Value	Estimate	95% CI	*p*-Value
Time 1 versus time 0	70.4	(48.9, 92.0)	<0.001 *	59.7	(41.9, 77.4)	<0.001 *
Time 2 versus time 0	63.7	(35.5, 91.9)	<0.001 *	64.2	(40.8, 87.6)	<0.001 *
Time 2 versus time 1	−6.7	(−30.9, 17.4)	0.789	4.5	(−15.3, 24.4)	0.853

* indicates statistical significance.

**Table 3:** Proximodistal and dorsopalmar extents and the shape of hypoattenuating lesions in the sagittal ridge of the third metacarpal bone in Thoroughbred racehorses measured at three subsequent computed tomographic examinations at intervals of approximately six months. n—number of limbs, CI—confidence interval.

		Time 0 (n = 80)	Time 1 (n = 62)	Time 2 (n = 46)
**Proximodistal extent (mm)**	Median	5.3	4.3	4.0
	95% CI	4.3–11.7	3.6–12.3	2.9–14.5
**Dorsopalmar extent (mm)**	Median	2.1	1.7	2.1
	95% CI	0.7–1.9	0.5–1.6	0.5–2.5
**Shape**				
Proximodistally elongated		12	6	5
Extending towards trabecular bone		16	10	5
Depression		1	1	1
Indentation		2	1	1
Subtle		2	4	2

## Data Availability

Anonymised raw data are available upon reasonable request.
